# Breast Tumour Kinase (Brk/PTK6) Contributes to Breast Tumour Xenograft Growth and Modulates Chemotherapeutic Responses In Vitro

**DOI:** 10.3390/genes13030402

**Published:** 2022-02-24

**Authors:** Rajpal S. Burmi, Gary M. Box, Umar Wazir, Haroon A. Hussain, Julie A. Davies, William J. Court, Suzanne A. Eccles, Wen G. Jiang, Kefah Mokbel, Amanda J. Harvey

**Affiliations:** 1 Centre for Genome Engineering and Maintenance, Institute for Health Medicine and Environments, Brunel University London, Uxbridge UB8 3PH, UK; rajpal.burmi@gmail.com (R.S.B.); haroon.hussain@brunel.ac.uk (H.A.H.); julie.davies@ge.com (J.A.D.); 2 The Cancer Research UK Cancer Therapeutics Unit, McElwain Laboratories, The Institute of Cancer Research, Sutton SM2 5NG, UK; gary.box@icr.ac.uk (G.M.B.); will.court@icr.ac.uk (W.J.C.); sue.eccles01@icr.ac.uk (S.A.E.); 3 The London Breast Institute, Princess Grace Hospital, London W1U 5NY, UK; umar.wazir@rcsed.ac.uk (U.W.); kefah.mokbel@hcahealthcare.co.uk (K.M.); 4 Cardiff China Medical Research Collaborative, Cardiff University School of Medicine, Heath Park, Cardiff CF14 4XN, UK; jiangw@cardiff.ac.uk

**Keywords:** Brk/PTK6, ALT-PTK6, breast cancer, chemotherapy, kinase, prognosis

## Abstract

Breast tumour kinase (Brk/PTK6) is overexpressed in up to 86% of breast cancers and is associated with poorer patient outcomes. It is considered a potential therapeutic target in breast cancer, even though the full spectrum of its kinase activity is not known. This study investigated the role of the kinase domain in promoting tumour growth and its potential in sensitising triple negative breast cancer cells to standard of care chemotherapy. Triple negative human xenograft models revealed that both kinase-inactive and wild-type Brk promoted xenograft growth. Suppression of Brk activity in cells subsequently co-treated with the chemotherapy agents doxorubicin or paclitaxel resulted in an increased cell sensitivity to these agents. In triple negative breast cancer cell lines, the inhibition of Brk kinase activity augmented the effects of doxorubicin or paclitaxel. High expression of the alternatively spliced isoform, ALT-PTK6, resulted in improved patient outcomes. Our study is the first to show a role for kinase-inactive Brk in human breast tumour xenograft growth; therefore, it is unlikely that kinase inhibition of Brk, in isolation, would halt tumour growth in vivo. Breast cancer cell responses to chemotherapy in vitro were kinase-dependent, indicating that treatment with kinase inhibitors could be a fruitful avenue for combinatorial treatment. Of particular prognostic value is the ratio of ALT-PTK6:Brk expression in predicating patient outcomes.

## 1. Introduction

Breast tumour kinase, also known as protein tyrosine kinase 6 (Brk/PTK6) is overexpressed in a high proportion of invasive breast cancers, and represents a possible target for therapeutic intervention [1,2]. In vitro studies have shown that Brk expression increases breast cancer cell proliferation [3], migration [4,5,6], and protects the cells from the induction of autophagic and apoptotic cell death [1,7].

Brk involvement in regulating ErbB (erythroblastic leukemia viral oncogene B) receptor signaling is its most well-described role in breast tumorigenesis. Brk is activated in response to ErbB receptor ligands such as EGF or heregulin and, as it associates with all 4 ErbB receptors, Brk has the potential to promote downstream signaling in response to ligand binding [2,8,9,10,11]. Increased activation of PI3-K/Akt, p190 RhoGAP, Rac GTPase, Erk5, p38 MAPK, and ARAP-1 have all been attributed to Brk activity in response to ErbB ligands, resulting in increased cell proliferation, cell cycle progression, and migration [2,11,12,13,14].

Brk also regulates processes involved in breast cancer progression in response to non-ErbB receptor ligands such as HGF, OPN, and IGF [6,7,15]. Research avenues away from unravelling Brk signaling have focussed on Brk effects in tumour development and treatment.

Despite the wealth of in vitro data and accompanying correlative studies from clinical samples, there are surprisingly few data regarding the potential for increased tumour growth associated with Brk activity. *Ptk6* (Brk) expression was shown to enhance the formation of murine ErbB2-induced tumours in cleared fat pads with a shorter latency period than ErbB2-only tumours [12], and both constitutively active and wild-type Brk promoted xenograft growth of MDA-MB-231 basal breast carcinoma cells [16]. However, there are no published data demonstrating kinase dependency in xenograft tumour development.

Xiang and colleagues showed that Brk also decreased the efficacy of the dual EGFR/HER2 inhibitor lapatinib [12]. Our previous work showed that Brk could, in some cellular contexts, affect the relative levels of the anti-apoptotic protein Bcl-x_L_ and its pro-apoptotic splice variant Bcl-x_S_ in favour of Bcl-x_S_ [1]. Given that Bcl-x_L_ has been linked with chemotherapeutic resistance to treatments such as doxorubicin [17], it was hypothesized that Brk expression could modulate tumour cell responses to doxorubicin and possibly other chemotherapeutic agents.

Here we studied whether kinase activity was underlying Brk’s growth-promoting effect in human tumour xenografts and breast cancer cell sensitivity to chemotherapeutic agents in vitro and examined whether alternative splicing of Brk had any impact on patient outcomes.

## 2. Materials and Methods

### 2.1. Cell Culture

T-47D breast cancer cells were cultured in RPMI-1640 supplemented with 10% foetal bovine serum (FBS), 2 mM glutamine, 100 U/mL penicillin, and 100 μg/mL streptomycin in a humidified atmosphere of 5% CO_2_ in air at 37 °C. GI101 cells were cultured in the same medium supplemented with 5 μg/mL insulin.

Brk-transfected cell lines were generated as previously described, with vectors encoding wild-type (WT) or kinase-inactive Brk. Kinase activity was abolished through the mutation of Lysine (K) 219 to Methionine (M) and the protein designated Brk (KM) [1,3]. Cells were maintained in 400 μg/mL G418 (Invitrogen, Paisley, UK).

Paclitaxel-resistant T-47D cells (a gift from Dr Helen Coley at the University of Surrey) were developed by exposing the cells to incrementally increased doses of paclitaxel with each successive passage as previously published for MCF7 cells [18]. Once a resistant phenotype was established, the cells were cultured in RPMI-1640 as above with 4 nM paclitaxel (for resistant cells) or a drug vehicle (for parental cells), at every alternate passage.

### 2.2. RNA Interference

RNA interference protocols were carried out using 120 nM Control non-targeting or PTK6-targeting on-TARGET-PLUS SMART POOL siRNA (Dharmacon, Thermofisher, Hemel Hempsted, UK) and Oligofectamine (Invitrogen, Paisley, UK). A two-hit transfection strategy at 24 h-intervals was employed as previously described [1].

Suppression was confirmed by Western blotting of whole cell protein lysates.

### 2.3. Human Tumour Xenografts

All studies were carried out according to the National Cancer Research Institute (NCRI) guidelines [19].

Xenograft tumours were established by the inoculation of 5 × 10^6^ MDA-MB-157cells, transfected with the empty vector (pRcCMV) or vector encoding a cDNA for either wild-type Brk (WT-Brk) or kinase-inactive Brk (KM-Brk) into the 3rd anterior mammary fat pad of female CrTac:NCr-*Fox-1(nu)* athymic mice. Tumour growth was measured by calipers and volumes were calculated over 118 days. The animals were housed according to institutional and British Home Office regulations in filter boxes in laminar flow cabinets. Food and water, along with all equipment and reagents, was sterilized.

### 2.4. Dose Response Determinations

Cells were seeded at 15 × 10^3^ cells per well into 96-well plates and allowed to adhere overnight. Following serial dilution in vehicle (DMSO or Ethanol) to produce a series of 1000-fold stocks, the drugs were diluted 1:1000 in culture medium and added to the plate at final concentrations between 0.01 and 10 μM. A 1:1000 dilution of vehicle was used as a control and all wells (control and drug-treated) contained the same volume of vehicle. Drugs were purchased from Sigma- Aldrich (Poole, UK) and Compound 4f was purchased from Andreas Hilgeroth, Martin-Luther-University Halle-Wittenberg, Universitätsplatz 10, 06108 Halle (Saale)Relative cell numbers were determined after 72 h of drug treatment by MTT assay (Sigma-Aldrich, Poole, UK).

### 2.5. Western Blot

Whole cell lysates were generated by lysing cells in 2 × SDS-PAGE lysis buffer and proteins were separated by SDS-PAGE on a 10% SDS-polyacrylamide gel. Following electro-blotting in 1× Towbin buffer onto nitrocellulose membranes, membranes were blocked in 5% non-fat dried milk in TBS/0.1% Tween. Membranes were then incubated overnight with a primary antibody diluted 1:10 in 5% BSA in TBS/0.1% Tween for Brk (ICR-100) [10] and 1:1000 for β-actin (Abcam, Cambridge, UK) in 5% non-fat milk in TBS/0.1% Tween. Membranes were washed and proteins visualised with an appropriate secondary antibody, conjugated to horseradish peroxidase (Dako, Cambridge, UK) and chemiluminescent substrate (Pierce, Thermofisher, Hemel Hempsted, UK).

### 2.6. Human Breast Tumour Samples

Breast cancer tissues (n = 127) and normal background tissue (n = 33) were collected and stored at −80 °C until the commencement of the study. The patient cohort has been part of a number of completed and on-going studies, and the clinicopathological data describing the patient cohort is further described by Wazir et al. [20]. The Kaplan–Meier survival graphs and statistical analysis were generated using SPSS26. An RUC model was used to generate a cut-off point for grouping the patients into high and low expression groups for survival analysis. Tissue samples were used with informed consent and ethical approval for the experiments were obtained as per institutional guidelines and ethical approval as detailed by Wazir et al. [20].

Statistical analysis was determined at multiple levels using a number of statistical tests, including Logrank (Mantel Cox) for significant difference between high and low PTK6 expression in relation to overall survival at later time points with a Breslow (generalized Wilcoxon) test showing significant difference at the earlier time points. The 100 Tarone–Ware test was used to further assess the distribution of overall survival with assumptions that there is an identical distribution as well as equal variance.

### 2.7. Tissue Processing, RNA Extraction, and cDNA Synthesis

Approximately 10 mg of cancerous tissue and normal mammary tissue was homogenised and RNA was extracted using an RNA extraction kit from Abgene (Epsom Surrey UK). The concentration of RNA was determined using a UV spectrophotometer (Wolf Laboratories, York, UK) to ensure adequate amounts of RNA for analysis. Reverse transcription was carried out using a reverse transcription kit (AbGene, Epsom, Surrey, UK) with an anchored olig (dT) primer using 1 g of total RNA in a 96-well plate to produce cDNA. The quality of cDNA was verified using β-actin primers (primers 5′-ATGATATCGCCGCGCTCGTC-3′ and 5′-CGCTCGGTGAGGATCTTCA-3′).

### 2.8. Real-Time PCR

Quantitative analysis of transcripts of the cDNA library of breast cancer and normal mammary tissues were determined using real-time quantitative PCR based on Amplifluor technology with PCR primers designed using Beacon Designer software (Premier Biosoft International Ltd., Pal Alto, CA, USA) (Table 1). To each of the reverse primers, a Z-sequence (5′actgaacctgaccgtaca′3) that was complementary to the Uniprimertm (Millipore) was added and the reaction was carried out using StepOnePlus thermocyclers under the following conditions: 94 °C for 10 min and 55 cycles of 94 °C for 10 s, 55 °C for 30 s, and 72 °C for 15 with detection of fluorescence signal at the annealing step of each cycle. The levels of each transcript Expression of PTK6 transcripts were normalised against cytokeratin 19 (CK19).

### 2.9. Statistical Analysis

Numerical data was analysed using the Excel spreadsheet software and significance was determined by Student’s *t*-test. *p* < 0.05 was considered significant.

## 3. Results

### 3.1. Brk Promotes Breast Xenograft Formation

One of the initial aims of this study was to examine the potential of Brk to promote breast tumour growth, independent of exogenous influences such as HER2 expression, to eliminate the effect of HER2 in tumour growth, as most Brk expressing tumours are HER2 negative, and to establish whether the kinase function of Brk was required.

**Figure 1:**
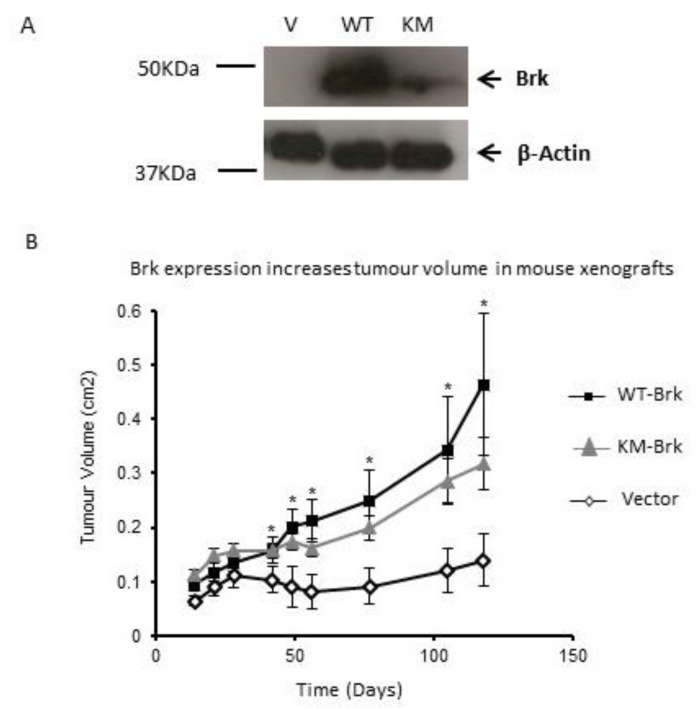
Brk promotes breast tumour xenograft growth (**A**) MDA-MB-157 cells were stably transfected with either the pRc/CMV vector (Vector), or a vector encoding wild-type (WT Brk) or kinase-inactive Brk (KM Brk), and the expression was confirmed by Western blotting with β-Actin expression as a loading control. (**B**) Cells were injected into the mammary fat pad of female CrTac:NCr-Fox-1(nu) athymic mice and tumour volume was calculated from the measurement of two perpendicular diameters over 118 days. Mean tumour volume (n = 10) +/− SEM is plotted. * indicates *p* < 0.05 (student’s *t*-test, WT-Brk compared to the vector control).

To investigate whether Brk promotes tumour growth, Brk-negative MDA-MB-157 cells, stably transfected with either vector alone, a vector encoding wild-type Brk or kinase-inactive Brk, were injected into mammary fat pads of female CrTac:NCr-*Fox-1(nu)* athymic mice. Brk expression in MDA-MB-157 was confirmed by Western blotting (Figure 1A). Tumours were allowed to establish and their size was monitored up to 118 days post-injection. Parental MDA-MB-157 cells established small tumours that did not grow above 0.15 cm^2^ within the time-frame of the study.

The presence of wild-type Brk (WT-Brk) caused a significant increase in the size of tumours compared with the control vector-transfectants (Vector) (*p* < 0.05). Interestingly, kinase-inactive Brk (KM-Brk) also induced a significant increase in tumour volume compared with the vector-only cells (*p* < 0.05) at a rate that was only slightly less than those induced by wild-type Brk. This difference in size between tumours with KM-Brk and WT-Brk was not statistically significant (*p* > 0.05), suggesting that an active kinase domain is not required for xenograft tumour progression (Figure 1B). This opens new questions about the potential of the other domains within Brk that potentiate this growth.

### 3.2. Brk Reduces Breast Cancer Cell Susceptibility to Chemotherapeutic Agents In Vitro

As well as investigating Brk’s influence on tumour growth, we were also interested in whether Brk affected tumour cell responses to standard of care chemotherapeutic agents.

**Figure 2:**
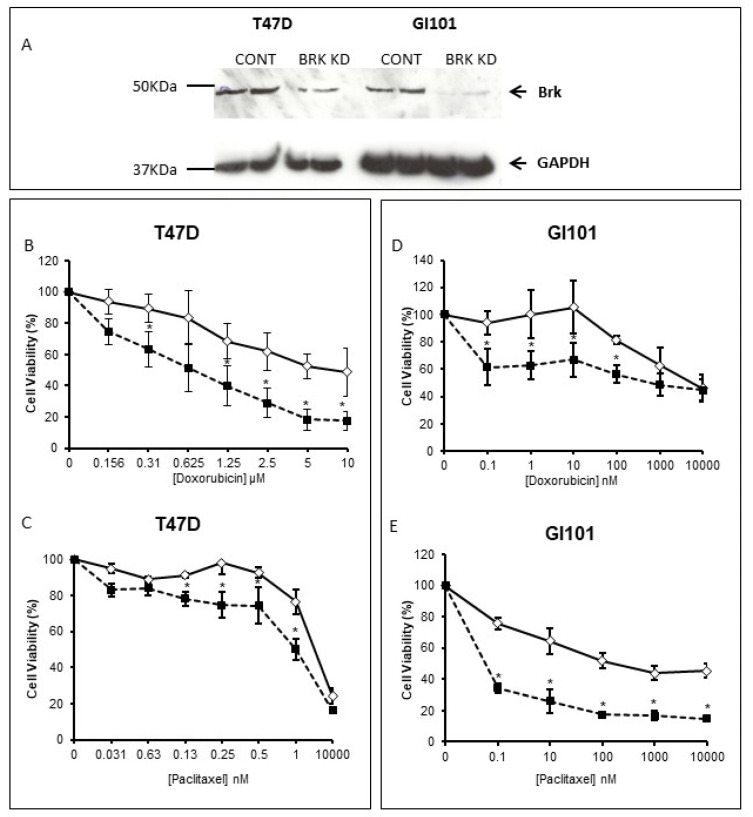
Brk moderates cell responses to chemotherapeutic agents. Brk levels were suppressed in T47D and GI101 cells by RNAi (Brk KD) and suppression was confirmed by Western blotting (**A**); the cells were seeded and allowed to adhere overnight in 96-well plates. Cells were treated with either doxorubicin (**B**,**D**) or Paclitaxel (**C**,**E**) for 72 h. Cell viability was determined by MTT assay and expressed as a percentage of the control (vehicle only) wells. The mean % survival (+/− SD) of a representative experiment is plotted with the control (transfected with no-target siRNA) in open diamonds and Brk-suppressed cells in closed squares; * indicates *p* < 0.05.

The human breast carcinoma cell line T-47D was tested for its responses to a number of chemotherapeutic agents, including doxorubicin and paclitaxel. Suppression of Brk by RNAi (Figure 2A) appeared to sensitise cells to both doxorubicin and Paclitaxel (Figure 2B,C), with the greatest effect seen with doxorubicin.

When Brk levels were suppressed in the breast cancer cell line GI101 (Figure 2A), the cells again became more susceptible to the effects of paclitaxel or doxorubicin (Figure 2D,E) compared with cells transfected with the control siRNAs. The percentage survival of GI101 cells treated with doxorubicin was significantly reduced at all doses tested between 0.1 and 100 nM, and at all doses of paclitaxel tested (0.1–10 μM).

In addition, T-47D cells that had been generated to develop resistance to paclitaxel (Tax-R) had elevated Brk levels compared with parental (Par) drug sensitive controls (Supplementary data Figure S1).

**Figure 3:**
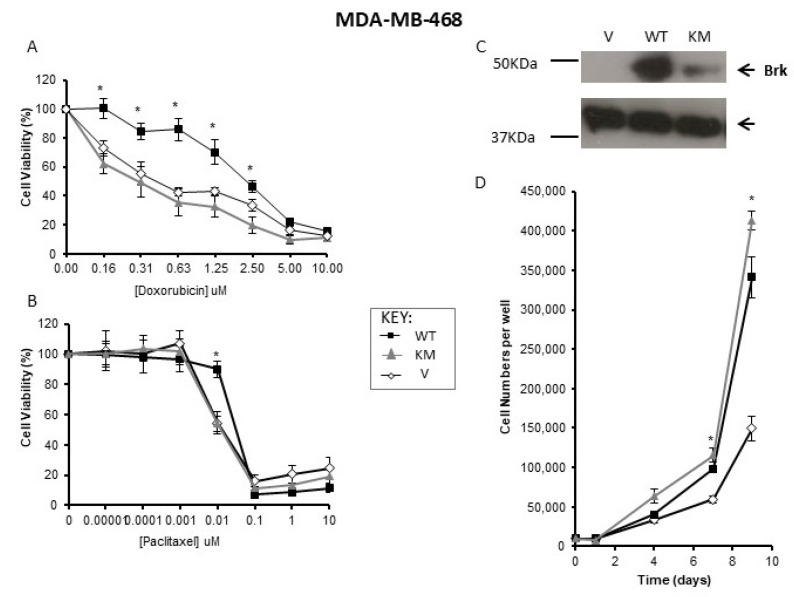
Brk’s effects on drug responses are dependent on kinase function and not proliferation. MDA-MB-468 cells, stably transfected with empty vector (V) or constructs expressing either wild-type (WT) or kinase-inactive Brk (KM), were seeded and allowed to adhere overnight in 96-well plates. Cells were treated with either (**A**) doxorubicin (upper panel) or (**B**) paclitaxel (lower panel) for 72 h. Cell viability was determined by MTT assay and expressed as a percentage of the control (vehicle only) wells. The mean % survival (+/− SD) of a representative experiment is plotted; * indicates *p* < 0.05. (**C**) Whole cells lysates were analysed for Brk expression by Western blotting with β-Actin expression as a loading control. (**D**) Cells were seeded into 24 well plates and replicate wells counted over 10 days.

To compare the effects of wild-type and kinase-inactive Brk individually on cell sensitivity to chemotherapy agents, a Brk-negative cell line, MDA-MB-468, was selected for transfection. When wild-type Brk was expressed in MDA-MB-468, cells became more resistant to the effects of doxorubicin, but not paclitaxel. Parallel transfections with KM-Brk were also carried out, but this did not affect cellular responses to paclitaxel or doxorubicin compared with vector-only control transfectants (Figure 3A,B). Expression levels were confirmed by Western blotting (Figure 3C).

To confirm that differences in cell growth in response to doxorubicin were not due to an altered proliferative capacity of cells with WT-Brk compared with KM-Brk, the effects of WT-Brk and KM-Brk on proliferation were investigated. Both WT-Brk and KM-Brk promoted the growth of MDA-MB-468 cells under standard culture conditions (Figure 3D), with the KM-Brk-transfected cells reaching higher cell numbers than those transfected with WT-Brk (*p* < 0.05).

Taken together these data suggest that the effects of Brk in mediating cell responses to chemotherapy agents require kinase activity and are not related to proliferative capacity.

### 3.3. Brk Inhibition in Triple Negative Breast Cancers

Due to their lack of receptors/molecular targets, triple negative breast cancers (TNBCs) have more limited treatment options than those that express ER and PR or overexpress HER2 and EGFR, which can be used as targets for therapy.

**Figure 4:**
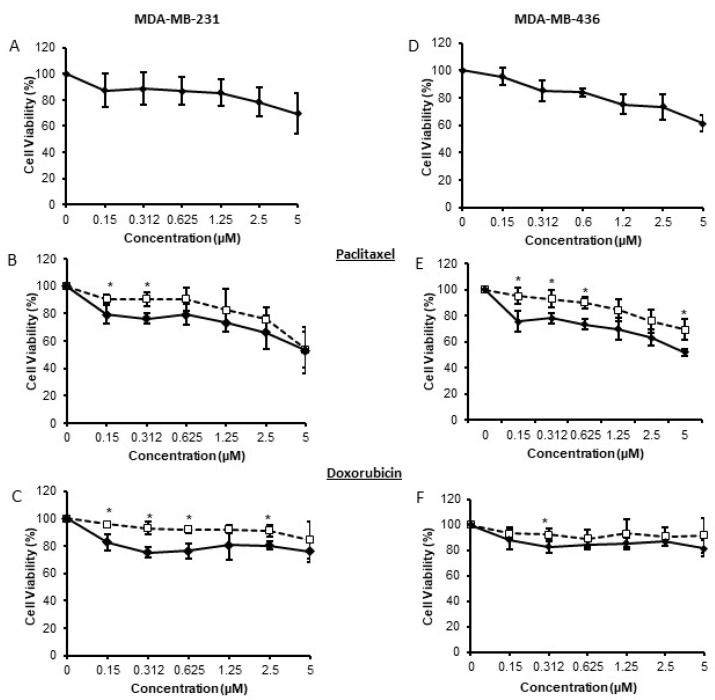
Inhibition of Brk’s kinase function increases cell sensitivity to chemotherapeutic agents. Triple negative MDA-MB-231 and MDA-MB-436 cells were seeded and allowed to adhere overnight in 96 wells plates. They were then treated with the Brk inhibitor Compound 4f alone (**A**,**D**) or in combination with paclitaxel (**B**,**E**) or doxrubicin (**C**,**F**) over a range of concentrations (0–5 µm). In B–F, open squares represent cells treated with doxorubicin or paclitaxel only; closed diamonds represent cells treated with both 4f and the chemotherapeutic agent. Cell viability was determined by MTT assay and expressed as a percentage of the control (vehicle only) wells. The mean % survival (+/− SD) of n = 3 experiments is plotted; * indicates *p* < 0.05.

Given that TNBCs express *ptk6* (Brk) (Supplementary Figure S2), that *ptk6* (Brk) expression is linked to poorer prognosis in TNBC [21], and Brk has potential as a therapeutic target [22], the effect of Brk kinase inhibition on cell viability was investigated. Inhibition of Brk with the kinase inhibitor compound 4f [23,24] resulted in significant, but very modest, decreases in cell viability over the dose range used (Figure 4A,D). The effects of 4f were determined by Western blotting (supplementary Figure S3).

Due to the current absence of molecular targets, many patients with TNBC will be treated with chemotherapy, but patients frequently relapse due to intrinsic or acquired resistance to the drugs. The modest decreases in cell viability suggest that inhibition of Brk is unlikely to be a mono-therapy. However, there may be potential to combine Brk inhibition with current standard of care therapeutics such as doxorubicin and paclitaxel. Therefore, the effect of 4f on TNBC cell responses to paclitaxel and doxorubicin was examined.

Co-treatment with compound 4f resulted in a modest, but significant, increase in responsiveness to doxorubicin and paclitaxel at the lower doses (nanomolar) in both MDA-MB-231 and MDA-MB-436 cells (Figure 4B,C,E,F).

These data suggest that the inhibition of Brk kinase function could improve triple negative breast cancer cell responses to standard of care chemotherapeutic agents.

### 3.4. Impact of Brk Splice Variants on Patient Outcomes

**Figure 5:**
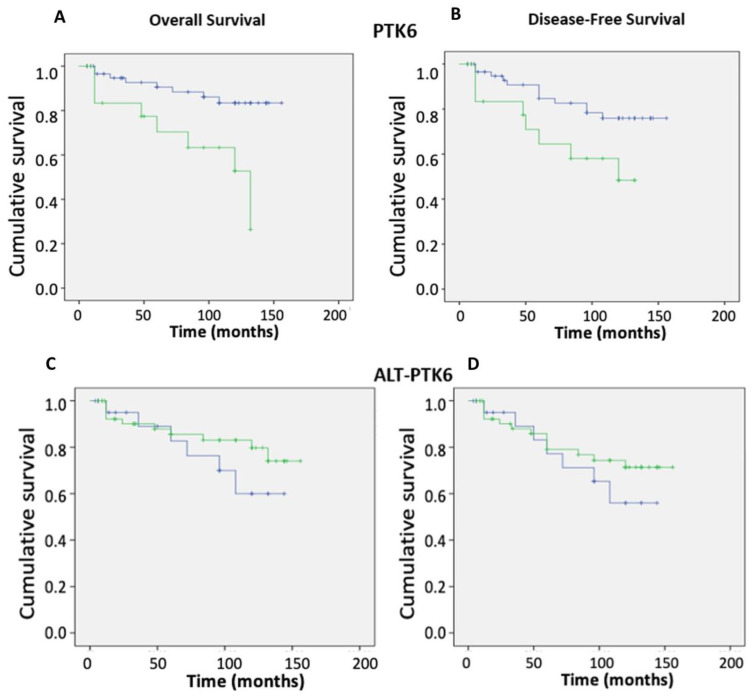
Effect of *ptk6* transcripts on patient outcomes. *Ptk6* transcript expression was determined in breast cancer tissue (n = 127) in relation to the normal background tissue (n = 33) using real-time qPCR and correlated with conventional clinic-pathological parameters and clinical outcomes. The Kaplan–Meier curves show high *ptk6* expression (green) and low *ptk6* (blue) levels in relation to (**A**) overall and (**B**) disease-free survival over time, as well as ALT-TPK6 in relation to (**C**) overall and (**D**) disease-free survival over time, where high expression is again in green and low expression is in blue. The statistical analysis is shown in the Supplemental Tables S1–S4.

*ptk6* mRNA itself is alternatively spliced with the alternate splice variant encoding a truncated protein that lacks the kinase domain and SH2 domains [25]. Given that we have previously shown that *ptk6* (Brk) expression has a negative impact on patient outcomes [21], but others have shown Brk to be a positive prognostic indicator, the impact of each isoform on breast cancer patient outcomes was examined. As previously reported, high expression of *ptk6* (Brk) was significantly associated with an increasing tumour (TNM) stage (*p* = 0.02) and resulted in reduced overall and disease-free survival (Figure 5A,B).

**Figure 6:**
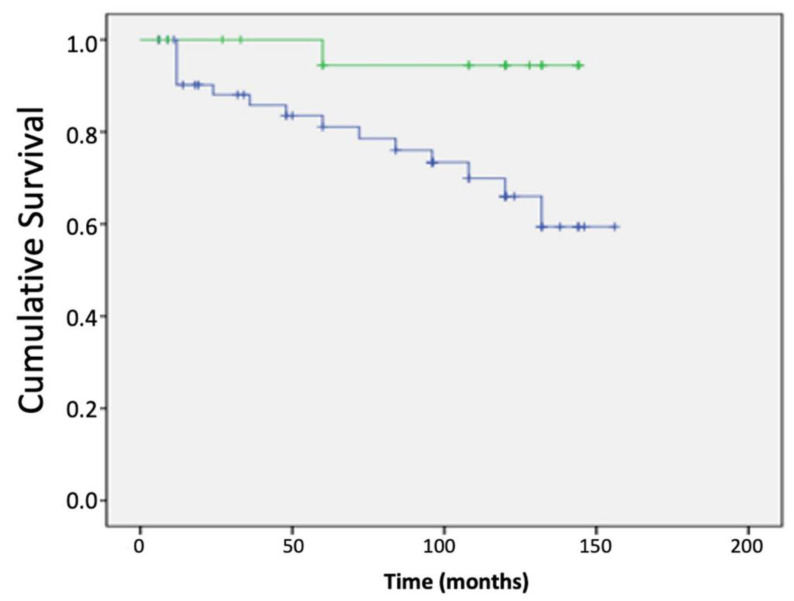
High ALT-PTK6: *ptk6* ratio results in favourable overall survival. *ptk6* transcript expression was determined in breast cancer tissue (n = 127) in relation to the normal background tissue (n = 33) using real-time qPCR and correlated with conventional clinic-pathological parameters and clinical outcomes. The Kaplan–Meier curves show high ALT-PTK: *ptk6* ratios (green) and low ALT-PTK: *ptk6* ratios (blue) levels in relation to overall survival. The statistical analysis is shown in the Supplemental Table S5.

However, the opposite was found for the alternatively spliced isoform ALT-PTK6, where high expression resulted in improved overall and disease-free outcomes (Figure 5C,D). Interestingly, the relative expression of the two isoforms was found to have prognostic significance as a high ratio of ALT-PTK6 expression compared with full length *ptk6 transcript* also resulted in improved overall survival (Figure 6). When receptor status was taken into account, a high ratio of *ptk6*/ALT-PTK6 transcripts was found in ER-ve tumours (Table 2).

## 4. Discussion

The aims of this study were to determine whether the kinase activity of Brk was fundamental for xenograft tumour development, and to investigate a proposed role for Brk in mediating cellular responses to chemotherapeutic agents.

Previous studies have indicated a role for Brk in tumour development alongside ErbB2 overexpression in an immortalized, mouse mammary cell line that retains normal morphology and function in vitro [12,26]. Miah and colleagues (2012) also demonstrated that both wild-type and constitutively active Brk promoted xenograft tumour formation [16]. Our study is the first to show a role for kinase-inactive Brk in human breast tumour xenograft growth. This was striking, but could be interpreted in terms of the multiple roles that Brk has in the processes underlying tumour development [27]. Many of these, for example tumour cell proliferation and migration, do not always require kinase activity [3,6]. In addition, abnormal expression of β-catenin is reported to be one of the mechanisms underlying breast tumorigenesis [28], and Brk is known to regulate β-catenin in a kinase-independent manner [29]. Interestingly, membrane-targeted Brk activated β-catenin/TCF-regulated transcription [29], and it is the membrane-localisation that affords Brk its oncogenic properties [30]. It remains to be explored which other Brk domains underlie the xenograft tumour growth, and whether new therapeutics could be developed to target these domains and their substrate interactions.

Our findings contradict those of Jiang and colleagues who reported that inhibiting Brk’s kinase function resulted in decreased xenograft growth [31]. It should be noted that the in vivo experiments carried out by Jiang used murine pro BA/F3 B lymphocyte cells, so caution should be exerted when extrapolating their findings to breast cancer.

Brk expression also appeared to regulate breast cancer cell responses to the chemotherapeutic agents paclitaxel and, especially, doxorubicin (Figure 3). As a mitotic spindle regulator, paclitaxel inhibits cell division and promotes cell death by stabilising microtubules in the G2/M phase of the cell cycle [32]. Doxorubicin, on the other hand, inhibits topoisomerase II, and could be predicted to have a greater impact on cell cycle progression from S-phase. Interestingly, previously published data show that Brk suppression results in a decrease in BrdU incorporation in T-47D cells. Parallel experiments examining cell cycle phase showed an increase in the percentage of cells in S-phase when Brk levels were reduced (unpublished findings), suggesting that, although BrdU incorporation decreased, Brk-suppression cells entered S-phase, which may make them more susceptible to doxorubicin than paclitaxel. This could account for the apparent difference in the magnitude of responses to doxorubicin and paclitaxel when Brk levels were decreased.

The observed regulation of the responses to chemotherapeutic drugs appeared to be dependent on the kinase activity of Brk (Figure 3). Consistent with our previously published data [3], MDA-MB-468 cells transfected with kinase-active Brk proliferated at a slightly higher rate to those transfected with wild-type Brk (Figure 3C). This suggested that the effect of *ptk6* (Brk) expression on drug responses is not an artefact of an increased proliferative response, and is indeed reliant on a functional kinase. Inhibition of Brk by 4f resulted in a significant, but modest, increase in sensitivity to the chemotherapy agents (Figure 4). The effects seen were weaker than those observed when Brk expression was suppressed by RNAi (Figure 2). This could be explained by the fact that Brk has kinase-independent functions, especially in relation to cell proliferation [3]. It is also possible that the inhibitor, 4f, is not very potent and a new design of an inhibitor will address this [31]. The effects of 4f on downstream targets were also very slight (Figure S3). Nonetheless, these data, in conjunction with those from Jiang, provide proof of concept that suppressing Brk kinase activity may enhance chemotherapeutic responses, independent of any effects on tumour progression, and will provide an interesting avenue for future research.

The alternatively spliced variants of Brk had opposing effects on patient outcomes. As expected, based on our previous studies [21], the full length *ptk6* transcripts had a negative impact on both overall and disease-free survival. This contrasts markedly with the impact of the ALT-PTK6 variant, which had a positive impact on patient outcomes (Figure 5). Given the truncated isoform lacks both kinase and SH2 domains [25], it is proposed that it acts as a competitive inhibitor of full length Brk [33]. Of particular note is the finding that high PTK6/ALT-PTK6 ratios were associated with ER-ve tumours, i.e., those where treatment options are more limited. Manipulating the expression of the isoforms so that the relative ratio is pushed more towards ALT-ptk6 may have clinical benefit in the longer term.

We, and others, have suggested for some time that inhibiting Brk may be of therapeutic benefit to breast cancer patients but, due to the kinase-independent functions of Brk, this possibility has not been widely pursued. The availability of Brk selective/specific kinase inhibitors remains limited, although there is recent progress in this area with the development of compounds, such as 4f, and the identification of extracts with anti-Brk properties. It is becoming increasingly apparent that targeting Brk with kinase inhibitors in isolation may have clinical limitations, and future Brk-directed therapies may need to focus on the reduction of Brk expression or disruption of protein–protein interactions for therapeutic benefit to be fully realized. This is supported by our data on the xenograft, in which we see growth regardless of the kinase activity of Brk, suggesting that other domains could underlie this growth and could be explored to target Brk alternative functions in the future. Nevertheless, the data in this report suggest that, as part of a multi-drug, combination therapy-based approach, targeting Brk with kinase inhibitors would still be of therapeutic value, especially at lower doses. This approach could have particular significance in Brk-positive, triple negative breast cancers, as they are intrinsically less sensitive to conventional chemotherapy agents and current targeted therapies are of no benefit due to the negative receptor status of the tumours.

## 5. Conclusions

This study is the first to show a role for kinase-inactive Brk in human breast tumour xenograft growth; therefore, it is unlikely that kinase inhibition of Brk, in isolation, would halt tumour growth in vivo. However, we found that breast cancer cell responses to chemotherapy in vitro were kinase-dependent, indicating that treatment with kinase inhibitors could be a fruitful avenue for combinatorial treatment. Determining the relative ratios of Brk isoforms may be needed to fully understand the prognostic significance of Brk expression and/or the implications of any combination therapies.

## Figures and Tables

**Table 1:** Primers used in real-time PCR.

Gene	Forward Primer (5′-’3)	Reverse Primer (5′-’3)
PTK6V1	gttctttggctgcatctc	actgaacctgaccgtacattctcgctgaccctgat
PTK6V2	caactacctggccgaga	actgaacctgaccgtacagccctgtggtagttcaca

**Table 2:** Expression levels of *ptk6*, ALT-PTK6, and *ptk6*/ALT-PTK6 ratio in relation to hormone receptor status of breast cancer.

	HR Status		Transcript Level (Mean ± SD)	*p* Value ^a^
ALT-PTK6	ER	ER (−)	320.5 ± 107.0	0.15
		ER (+)	181.9 ± 68.9	
PTK6		ER (−)	4.6 ± 2.4	0.262
		ER (+)	3.0 ± 1.6	
PTK6/ALT-PTK6 ratio		ER (−)	10,842.3 ± 5207.3	0.042
		ER (+)	4.5 ± 4.5	
ALT-PTK6	HER2	HER2 (−)	274.1 ± 139.8	0.976
		HER2 (+)	266.4 ± 78.4	
PTK6		HER2 (−)	3.7 ± 1.8	0.796
		HER2 (+)	4.0 ± 1.9	
PTK6/ALT-PTK6 ratio		HER2 (−)	10,885.8 ± 7001.6	0.616
		HER2 (+)	8937.8 ± 4141.9	
ALT-PTK6	PR	PR (−)	306.1 ± 90.6	0.2
		PR (+)	168.1 ± 75.5	
PTK6		PR (−)	4.4 ± 2.0	0.314
		PR (+)	1.4 ± 0.4	
PTK6/ALT-PTK6 ratio		PR (−)	9437.09 ± 4479.549	0.565
		PR (+)	6507.15 ± 4462.946	

^a^ ANOVA test.

## Data Availability

No datasets were generated during this study.

## Supplementary Materials

The following supporting information can be downloaded at: https://www.mdpi.com/article/10.3390/genes13030402/s1. Figure S1: Brk is elevated in Taxol-resistant breast cancer cells. Parental (Parent) and Taxol-resistant (Tax-R) T47D cells were lysed in 2x SDS-PAGE loading buffer and Brk levels assessed by western blotting. Figure S2: Triple negative breast cancer (TBNC) cell lines express Brk. Western blot comparing the levels of Brk protein in MDA-MB-231 and MDA-MB-436 cells relative to other breast cancer cell lines. Figure S3: Effect of 4f on phosphorylation. MDA-MB-231 (left) and MDA-MB-436 (right) cells were treated with inhibitor 4f for the indicated time period and the effect on protein levels and phosphorylation determined by western blotting. Table S1: PTK6 overall survival. Table S2: PTK6 disease free survival. Table S3: ALT-PTK6 overall survival. Table S4: ALT-PTK6 disease free survival. Table S5: ALT-PTK6:PTK6 ratio and overall survival.
