# Supplementary material for: Breast Tumour Kinase (Brk/PTK6) Contributes to Breast Tumour Xenograft Growth and Modulates Chemotherapeutic Responses In Vitro
Source: Genes (Basel). 2022 Feb 24;13(3):402. doi: 10.3390/genes13030402 (PMC8950834; doi:10.3390/genes13030402)
Supplement: Supplementary file 1 [file genes-13-00402-s001.zip › genes-1591037-supplementary.pdf]

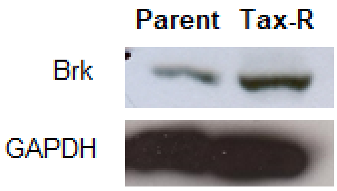

Supplemental Figure S1: Brk is elevated in Taxol-resistant breast cancer cells. Parental (Parent) and Taxol-resistant (Tax-R) T47D cells were lysed in 2x SDS-PAGE loading buffer and Brk levels assessed by western blotting.

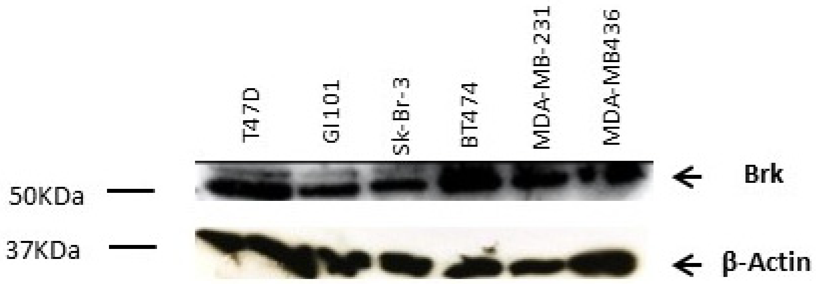

Figure S2. Triple negative breast cancer (TBNC) cell lines express Brk. Western blot comparing the levels of Brk protein in MDA-MB-231 and MDA-MB-436 cells relative to other breast cancer cell lines

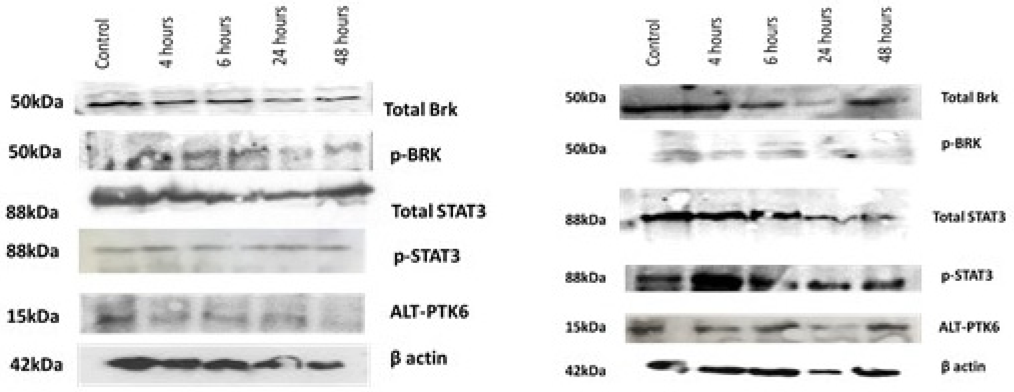

Figure S3. Effect of 4f on phosphorylation. MDA-MB-231 (left) and MDA-MB-436 (right) cells were treated with inhibitor 4f for the indicated time period and the effect on protein levels and phosphorylation determined by western blotting.

Supplemental Tables

Table S1: PTK6 overall survival

Overall Comparisons

	Chi-Square	df	Sig.
Log Rank (Mantel-Cox)	8.600	1	.003
Breslow (Generalized Wilcoxon)	6.339	1	.012
Tarone-Ware	7.213	1	.007

Test of equality of survival distributions for the different levels of VAR00019.

Means and Medians for Survival Time

VAR00019	Mean[a]				Median			
	Estimate	Std. Error	95% Confidence Interval		Estimate	Std. Error	95% Confidence Interval	
			Lower Bound	Upper Bound			Lower Bound	Upper Bound
.00	139.600	5.515	128.791	150.408	.	.	.	.
1.00	97.292	12.077	73.621	120.964	132.000	25.888	81.259	182.741
Overall	131.451	5.594	120.487	142.415	.	.	.	.

a. Estimation is limited to the largest survival time if it is censored.

Table S2: PTK6 disease free survival

Overall Comparisons

	Chi-Square	df	Sig.
Log Rank (Mantel-Cox)	4.517	1	.034
Breslow (Generalized Wilcoxon)	4.488	1	.034
Tarone-Ware	4.505	1	.034

Test of equality of survival distributions for the different levels of VAR00019.

Means and Medians for Survival Time

VAR00019	Mean[a]				Median			
	Estimate	Std. Error	95% Confidence Interval		Estimate	Std. Error	95% Confidence Interval	
			Lower Bound	Upper Bound			Lower Bound	Upper Bound
.00	132.496	6.200	120.343	144.648	.	.	.	.
1.00	92.813	11.417	70.436	115.191	120.000	.	.	.
Overall	125.752	5.965	114.061	137.443	.	.	.	.

a. Estimation is limited to the largest survival time if it is censored.

Table S3: ALT-PTK6 overall survival

Overall Comparisons

	Chi-Square	df	Sig.
Log Rank (Mantel-Cox)	1.153	1	.283
Breslow (Generalized Wilcoxon)	.639	1	.424
Tarone-Ware	.915	1	.339

Test of equality of survival distributions for the different levels of VAR00035.

Means and Medians for Survival Time

VAR00035	Mean[a]				Median			
	Estimate	Std. Error	95% Confidence Interval		Estimate	Std. Error	95% Confidence Interval	
			Lower Bound	Upper Bound			Lower Bound	Upper Bound
.00	114.411	10.272	94.277	134.545	.	.	.	.
1.00	133.032	6.747	119.808	146.256	.	.	.	.
Overall	130.029	5.876	118.512	141.547	.	.	.	.

a. Estimation is limited to the largest survival time if it is censored.

Table S4: ALT-PTK6 disease free survival

Overall Comparisons

	Chi-Square	df	Sig.
Log Rank (Mantel-Cox)	.734	1	.392
Breslow (Generalized Wilcoxon)	.329	1	.566
Tarone-Ware	.514	1	.474

Test of equality of survival distributions for the different levels of VAR00035.

Means and Medians for Survival Time

VAR00035	Mean[a]				Median			
	Estimate	Std. Error	95% Confidence Interval		Estimate	Std. Error	95% Confidence Interval	
			Lower Bound	Upper Bound			Lower Bound	Upper Bound
.00	109.935	10.599	89.161	130.708	.	.	.	.
1.00	126.414	7.312	112.082	140.746	.	.	.	.
Overall	123.929	6.255	111.668	136.190	.	.	.	.

a. Estimation is limited to the largest survival time if it is censored.

Table S5: ALT-PTK6:PTK6 ratio and overall survival

Overall Comparisons

	Chi-Square	df	Sig.
Log Rank (Mantel-Cox)	5.360	1	.021
Breslow (Generalized Wilcoxon)	4.730	1	.030
Tarone-Ware	5.071	1	.024

Test of equality of survival distributions for the different levels of VAR00014.

Means and Medians for Survival Time

VAR00014	Mean[a]				Median			
	Estimate	Std. Error	95% Confidence Interval		Estimate	Std. Error	95% Confidence Interval	
			Lower Bound	Upper Bound			Lower Bound	Upper Bound
.00	121.357	7.657	106.349	136.364	.	.	.	.
1.00	139.333	4.535	130.444	148.222	.	.	.	.
Overall	130.029	5.876	118.512	141.547	.	.	.	.

a. Estimation is limited to the largest survival time if it is censored.
